# Ellagic Acid Alleviates Imidacloprid-Induced Thyroid Dysfunction via *PI3K/Akt/mTOR*-Mediated Autophagy

**DOI:** 10.3390/toxics13050355

**Published:** 2025-04-29

**Authors:** Amina A. Farag, Mahmoud Mostafa, Reham M. Abdelfatah, Alshimaa Ezzat ELdahshan, Samar Fawzy Gad, Shimaa K. Mohamed, Mona K. Alawam, Aya Aly Elzeer, Nesma S. Ismail, Sally Elsharkawey, Haneen A. Al-Mazroua, Hatun A. Alomar, Wedad S. Sarawi, Heba S. Youssef

**Affiliations:** 1 Department of Forensic Medicine and Clinical Toxicology, Faculty of Medicine, Benha University, Benha 13518, Egypt; amina.farag@fmed.bu.edu.eg (A.A.F.); nesma.mohamed@fmed.bu.ed.eg (N.S.I.); sally.mansour@fmed.bu.ed.eg (S.E.); 2 Department of Pharmaceutics, Faculty of Pharmacy, Minia University, Minia 61519, Egypt; 3 Department of Pharmaceutics, Faculty of Pharmacy, Minia National University, New Minia 61768, Egypt; 4 Department of Pesticides, Faculty of Agriculture, Mansoura University, Mansoura 35516, Egypt; reham_2010@mans.edu.eg; 5 Department of Histology, Faculty of Medicine, Benha University, Benha 13518, Egypt; alshimaa.aldahshan@fmed.bu.edu.eg; 6 Department of Anatomy, Faculty of Medicine, Benha University, Benha 13518, Egypt; samar.gad@fmed.bu.edu.eg; 7 Department of Pharmacology and Toxicology, Faculty of Pharmacy, Helwan University, Cairo 11795, Egypt; shimaa_kamal@pharm.helwan.edu.eg; 8 Department of Physiology, Faculty of Medicine, Benha University, Benha 13518, Egypt; mona.alawam@fmed.bu.ed.eg (M.K.A.); heba.youssef@fmed.bu.edu.eg (H.S.Y.); 9 Department of Development of Animal Wealth, Faculty of Veterinary Medicine, Mansoura University, Mansoura 35516, Egypt; yoyo_elzeer@mans.edu.eg; 10 Department of Pharmacology and Toxicology, College of Pharmacy, King Saud University, Riyadh 11451, Saudi Arabia; halmazroua@ksu.edu.sa (H.A.A.-M.); hetalomar@ksu.edu.sa (H.A.A.); wsarawi@ksu.edu.sa (W.S.S.)

**Keywords:** thyroid dysfunction, ellagic acid, imidacloprid, autophagy, novasomes, network pharmacology

## Abstract

Imidacloprid (IMI) is a widely used insecticide known for its high selectivity toward insects. Ellagic acid (EA) is a plant-derived polyphenolic compound recognized for its therapeutic potential and favorable safety profile in the treatment of various diseases. This study aimed to evaluate the therapeutic effects of EA, formulated as novasomes (NOV), against IMI-induced thyroid dysfunction and to investigate the underlying mechanisms. Rats were divided into four equal groups: control, EA-NOV, IMI, and IMI + EA-NOV. Thyroid function was assessed by measuring free triiodothyronine (T3), free thyroxine (T4), and thyroid-stimulating hormone (TSH) levels. Thyroid tissues were examined to evaluate histopathological alterations, as well as to assess the oxidative/antioxidant pathway (Nrf2, SOD, TAC, MDA), inflammatory pathway (IL-1β, TNF-α, NF-κB), apoptotic pathway (*Bcl*, *BAX*), and autophagy pathway (*PI3K/Akt/mTOR*, *P53*, *Beclin-1*). Exposure to IMI resulted in impaired thyroid function, the upregulated gene expression of the *PI3K/Akt/mTOR* pathway, and downregulated *P53* expression. Additionally, immunohistochemical staining revealed Beclin-1-mediated autophagy, alongside increased apoptosis, oxidative stress, and elevated levels of inflammatory cytokines. Conversely, EA improved thyroid function and ameliorated histopathological alterations by enhancing autophagy-inducing pathways. Additionally, the alleviation of oxidative stress was evidenced by the increased immunohistochemical staining of Nrf2, which promoted the synthesis and activity of antioxidant enzymes and reduced apoptotic and inflammatory markers. This study proposes the use of EA as a potential protective, naturally occurring phytoceutical against IMI-induced thyroid dysfunction, primarily through the modulation of *PI3K/Akt/mTOR*-mediated autophagy.

## 1. Introduction

Neonicotinoid pesticides, a prevalent class of environmental pollutants, have dominated the agricultural sector since the late 1980s due to their effectiveness, affordability, and relatively low toxicity. However, their widespread environmental distribution poses significant health risks through various routes of exposure, including inhalation, dermal absorption, and ingestion via contaminated resources. Imidacloprid (IMI), a chlorinated derivative, is one of the most extensively used compounds in this class [1,2]. Zhao et al. characterized the urinary excretion profile of IMI and its degradation metabolites across a broad demographic spectrum [3]. This phenomenon of extensive use is primarily attributed to its high water solubility, low volatility, and prolonged degradation time in soil, which contribute to its persistence and eventual toxic effects on non-target organisms [2,4]. In addition, IMI residues can be detected in fruits and vegetables, posing potential health risks to humans [3].

Exposure to IMI has numerous adverse effects on various organs, including the hepatorenal and nervous systems [3]. A growing body of research has identified the thyroid gland as a vulnerable target for IMI-induced toxicity. Pesticides have been reported to disrupt thyroid function through multiple mechanisms, including interference with the synthesis, transport, and metabolism of thyroid hormones (THs) [5]. The thyroid gland is particularly susceptible to oxidative stress and inflammation [6]. In addition, THs regulate most bodily functions. Thyroid dysfunction is a serious condition associated with numerous metabolic disorders, including obesity, depression, diabetes mellitus, hypertension, and anxiety disorders [7].

An expanding body of research has identified oxidative stress and inflammatory responses as the principal molecular mechanisms underlying the toxic effects of IMI [8,9]. Contemporary experimental studies have elucidated the pivotal role of autophagy in mitigating cellular and tissue damage induced by IMI exposure. Autophagy is a vital intracellular process that facilitates the degradation and recycling of cellular components (CCs) through lysosomal digestion. This mechanism targets a diverse array of substrates, including segments of the plasma membrane, organelles—particularly mitochondria—protein aggregates, and cytoskeletal structures [10]. Autophagy promotes cell survival and viability by maintaining intracellular homeostasis. Notably, its activation prevents the accumulation of reactive oxygen species (ROS), thereby significantly reducing apoptosis and mitigating oxidative stress [11].

The *PI3K/Akt/mTOR* pathway plays a crucial role in the suppression of autophagy [12]. PI3K/Akt activates mTOR, which subsequently inhibits autophagy by preventing autophagosome formation [10]. Currently, the precise mechanisms underlying cellular damage induced by the IMI-mediated activation of the *PI3K/Akt/mTOR* signaling cascade remain unclear, warranting further in-depth investigation.

In recent years, natural products have been increasingly utilized in the treatment of various diseases due to their efficacy and safety profiles. Ellagic acid (EA) (4,4′,5,5′,6,6′-hexahydroxydiphenic acid 2,6,2′,6′-dilactone), a naturally derived polyphenolic compound, is primarily found in fruits such as pomegranates and berries. It is well documented for its antioxidant, anti-fibrotic, and anticancer properties [13,14,15]. EA has also been recognized for its anti-inflammatory properties, notably through the downregulation of TNF-α and NF-κB expression [16]. EA possesses a wide range of pharmacological properties, including cardioprotective, gastroprotective, anti-ulcer, hypolipidemic, neuroprotective, anticancer, hepatoprotective, and antidiabetic effects [17,18]. EA has also been documented to exert renoprotective effects through multiple molecular signaling pathways, including the stimulation of SIRT1 and modulation of the Nrf2/NF-κB signaling axis. These actions contribute to a reduction in oxidative stress and the suppression of the inflammatory cascade [19,20]. The relationship between thyroid function and EA has not been extensively studied. However, EA has demonstrated the ability to prevent oxidative damage to thyroid cells and protect against thyroid inflammation (thyroiditis) due to its antioxidant and anti-inflammatory properties. Moreover, it may counteract chronic inflammation associated with autoimmune thyroid disorders, such as Graves’ disease and Hashimoto’s thyroiditis. In addition, EA’s detoxifying effects may help neutralize certain environmental pollutants, heavy metals, and goitrogenic chemicals that interfere with iodine absorption or thyroid hormone synthesis [21,22,23]. Nonetheless, research on the potential protective mechanisms of EA in relation to thyroid function remains limited. Moreover, the clinical application of EA is limited by its poor water solubility, low bioavailability, susceptibility to photodegradation, and rapid metabolism—particularly when administered in dietary form [24]. To overcome these limitations, EA was incorporated into novasomes, a novel vesicular nanocarrier system. Novasomes offer several advantages, including high encapsulation efficiency, improved stability, sustained release, and enhanced cellular uptake [25]. These properties collectively improve the bioavailability and therapeutic efficacy of EA, positioning novasomes as a suitable and efficient delivery system for both toxicological and pharmacological studies.

This study investigates the toxicological effects of IMI exposure on the thyroid gland in rats, while simultaneously evaluating the potential protective role of EA-loaded novasomes in mitigating thyroid dysfunction. Furthermore, the study examines the functional impact of EA in counteracting oxidative stress, modulating inflammatory biomarkers, and influencing apoptotic pathways, with particular emphasis on its inhibitory effects on the *PI3K/Akt/mTOR* signaling cascade and its regulatory role in autophagy.

## 2. Materials and Methods

### 2.1. Materials

IMI (CAS No. 138261-41-3) was supplied by Bayer Crop Science, Germany. Span 60 and EA were purchased from Sigma-Aldrich (St. Louis, MO, USA). Cholesterol (Chol) was obtained from Fluka Chemicals (Raleigh, NC, USA), and oleic acid was procured from El-Nasr Company for Pharmaceutical Industries (Cairo, Egypt).

### 2.2. Network Pharmacology and Toxicology

#### 2.2.1. Acquisition of “Ellagic Acid”, “Imidacloprid”, and “Thyroid Dysfunction” Targets

The chemical structures of EA [26] and IMI [27], in SMILES format, were retrieved from the PubChem database (https://pubchem.ncbi.nlm.nih.gov/ (accessed on 26 April 2025)) (Supplementary Table S1). The pharmacokinetic properties and drug-likeness of EA were evaluated using the SwissADME tool [28].

The Comparative Toxicogenomics Database (CTD, http://ctdbase.org/ (accessed on 26 April 2025)) [29], SwissTargetPrediction (http://www.swisstargetprediction.ch/ (accessed on 26 April 2025)) [30], and SuperPred (https://prediction.charite.de/index.php (accessed on 26 April 2025)) [31] were explored to identify potential targets associated with EA. Potential targets of IMI were obtained from CTD [29], SuperPred [31], SwissTargetPrediction [30], and the Toxin and Toxin Target Database (T3DB, https://www.t3db.ca/ (accessed on 26 April 2025)) [32].

Disease-associated targets were extracted from DisGeNET (5), GeneCards (GC, https://www.genecards.org/ (accessed on 26 April 2025)) [33], and the Open Targets Platform (OTP, https://platform.opentargets.org/ (accessed on 26 April 2025)) [34]. The search terms “Thyroid Diseases” and “Thyroiditis” were used across all three databases, while “Abnormality of Thyroid Gland” was specifically used in the OTP. The UniProt Knowledgebase (https://www.uniprot.org/ (accessed on 26 April 2025)) [35] was employed to validate targets related to EA, IMI, and thyroid-associated diseases, using “*Homo sapiens*” as the selected species.

#### 2.2.2. Identification of Shared Targets and Construction of Protein–Protein Interactions

A Venn diagram platform (https://bioinfogp.cnb.csic.es/tools/venny/ (accessed on 26 April 2025)) [36] was used to analyze the overlapping targets of EA, IMI, and thyroid dysfunction. Conventional targets were then retrieved and introduced into the STRING database (https://www.string-db.org/ (accessed on 26 April 2025)) [37] for protein–protein interaction (PPI) network construction, with “*Homo sapiens*” set as the filtering parameter and a minimum interaction score threshold of 0.40.

#### 2.2.3. Network Construction and Examination

The PPI network was imported into Cytoscape 3.10.2 and filtered using the “Degree” algorithm to evaluate the interaction intensity among the core targets [38]. The intersecting targets were analyzed using ShinyGO (http://bioinformatics.sdstate.edu/go/ (accessed on 26 April 2025)) for Gene Ontology (GO) enrichment analysis [39]. To enhance the functional characterization of gene-related processes, GO analysis was conducted, classifying genes into three categories: CCs, biological processes (BPs), and molecular functions (MFs). Additionally, KEGG pathway enrichment analysis was performed using ShinyGO to elucidate the pharmacological effects of EA against IMI-induced thyroid dysfunction. A false discovery rate (FDR) threshold of <0.05 was considered statistically significant.

### 2.3. Preparation and Characterization of Ellagic Acid-Loaded Novasomes

The solvent injection method was employed to prepare EA-encapsulated novasomal systems [40,41]. In brief, the organic phase was prepared by dissolving Span 60, cholesterol, oleic acid, and EA in a weight ratio of 3:1.15:1:0.65 in 5 mL of absolute ethanol, and the mixture was maintained at a temperature of 60–70 °C. The preheated organic phase was then injected into water maintained at the same temperature (60–70 °C) using a 25 G syringe, while stirring at 1000 rpm in a closed system. After injection, the suspension was stirred continuously at the same temperature until the residual ethanol had completely evaporated. The EA-loaded novasomes were then stored at 4 °C until further analysis. To evaluate the novasomal systems, several parameters were assessed, including entrapment efficiency percentage (EE%), in vitro drug release, zeta potential (ZP), particle size (PS), transmission electron microscopy (TEM), and stability. Stability was assessed by storing the dispersed novasome samples at 4 °C for 30 days, during which the EE%, PS, ZP, and polydispersity index (PDI) were monitored.

### 2.4. Animals and Ethics

For this experiment, 24 adult male Albino rats (Rattus norvegicus), aged 8–10 weeks and weighing 180–210 g, were used. To ensure optimal environmental conditions, six rats were housed per decontaminated enclosure under controlled conditions, including a relative humidity of 55 ± 5%, a 12 h light/dark cycle, and a temperature of 23 ± 2 °C. Ethical approval for this study was obtained from the Research Ethics Committee of the Mansoura University Animal Care and Use Committee (Approval No. MU-ACUC (AGR.R.24.05.8)). All experimental procedures complied with the NIH Guide for the Care and Use of Laboratory Animals (NIH Publication No. 8023, revised 1978) and adhered to the ARRIVE guidelines.

#### 2.4.1. Experimental Design

Each rat was initially weighed and then randomly assigned to one of four experimental groups, with six animals per group. The animal groups and corresponding treatments were as follows:

Control group: rats were administered 1 mL of corn oil (the solvent for IMI) orally once daily for 4 weeks.

EA-NOV group: Rats received EA-loaded novasomes (10 mg/kg) via oral gavage once daily for 4 weeks. The dose of EA was selected based on its established antioxidant and protective effects in similar toxicological models [42,43].

IMI group: Rats received IMI at a dose of 45 mg/kg, dissolved in corn oil and administered via oral gavage, once daily for 4 weeks. This dose, equivalent to 1/10 of the reported oral LD_50_ (450 mg/kg), was selected based on studies by Mikolić and Karačonji (2018) and the WHO (2020) [44,45]. The LD_50_ value is consistent with that reported by Tomlin (2006) [46].

IMI + EA-NOV group: rats received oral EA-loaded novasomes (10 mg/kg) one hour prior to the oral administration of IMI (45 mg/kg), once daily for 4 weeks.

#### 2.4.2. Specimen and Serum Collection

On the 29th day, rats were anesthetized by inhalation of 1.9% diethyl ether (0.08 mL/L). Blood samples were collected using a sterilized fine glass capillary tube and transferred into vacutainer tubes. The samples were then centrifuged at 3000× *g* for 10 min, and the separated serum was stored at −20 °C for subsequent biochemical analysis. Following blood collection, the thyroid glands were excised and weighed. The relative thyroid weight (thyroid weight/body weight ratio) was calculated for each group. Each thyroid gland was divided into three portions: one for histopathological examination, another for the assessment of inflammatory markers and oxidative stress, and the final portion for storage at −80 °C for subsequent mRNA quantification using real-time PCR (RT-PCR).

#### 2.4.3. Biochemical Analyses

##### Assessment of Thyroid Function

Since free T3 and free T4 are a measure of the unbound, biologically active forms of THs and are less affected by external variables, they offer a clearer and more accurate assessment of thyroid function compared to total hormone levels [47]. The serum levels of free triiodothyronine (T3, Cat. No. MBS2000350) and free thyroxine (T4, Cat. No. MBS2000107) in rats were measured using enzyme-linked immunosorbent assay (ELISA) kits, following the manufacturer’s instructions provided with each kit. Thyroid-stimulating hormone (TSH, Cat. No. MBS729687) was quantified using a competitive ELISA method.

##### Estimation of Oxidative Stress Markers

Thyroid tissue homogenates were subjected to biochemical analysis to assess antioxidant activity and lipid peroxidation status. Malondialdehyde (MDA, Cat. No. SD 25 29), total antioxidant capacity (TAC, Cat. No. SD 25 13), and superoxide dismutase (SOD, Cat. No. SD 25 21) assay kits were obtained from BioDiagnostic (Giza, Egypt).

##### Estimation of Inflammatory Mediators

Thyroid tissue homogenates were analyzed using ELISA kits to quantify interleukin-1 beta (IL-1β) and tumor necrosis factor-alpha (TNF-α) levels. The IL-1β (Cat. No. RLB00) and TNF-α (Cat. No. RTA00) ELISA kits were purchased from R&D Systems (Minneapolis, MN, USA).

#### 2.4.4. Real-Time Quantitative PCR (qPCR) Analysis of mRNA Expression

##### RNA Extraction

Total RNA was isolated from thyroid tissue samples using the QIAamp RNeasy Mini Kit (Qiagen GmbH, Hilden, Germany), incorporating on-column DNase digestion to eliminate residual genomic DNA. The procedure began by mixing 200 μL of the sample with 600 μL of RLT buffer supplemented with 10 μL/mL of β-mercaptoethanol, followed by 10 min incubation at room temperature. Subsequently, an equal volume of 70% ethanol was added to the lysate, and RNA extraction was completed according to the QIAamp RNeasy Mini Kit total RNA protocol.

##### SYBR Green RT-PCR

A 20 μL reaction mixture was prepared for RT-qPCR analysis, consisting of 10 μL of 2× HERA SYBR Green RT-qPCR Master Mix (Willowfort, UK), 1 μL of 20× RT Enzyme Mix, 1 μL of each primer (20 pmol), 3 μL of nuclease-free water, and 5 μL of RNA template. The reactions were performed using a StepOne™ Real-Time PCR System at the Biotechnology Unit of the Animal Health Research Institute, Egypt. Primers were obtained from Metabion (Planegg, Germany), and their sequences are listed in Table 1.

##### Analysis of the SYBR Green RT-PCR Results

Amplification curves and CT values were analyzed using StepOne software. To evaluate gene expression differences among the RNA samples, CT values were normalized to those of the positive control group using the ΔΔCT method as described by Yuan et al. (2006) [48]. Relative gene expression levels were calculated using the following formula: 2^−ΔΔCT^.

#### 2.4.5. Histopathological Technique

At the end of the experiment, a small portion of fresh thyroid tissue was fixed in 10% formaldehyde overnight, followed by serial dehydration in ascending concentrations of ethanol and treatment with xylene. The tissue was then embedded in paraffin, and sections of 5 μm thickness were prepared using a microtome. To remove the paraffin, the sections were initially treated with xylene, followed by gradual rehydration through descending ethanol concentrations and a final rinse in water. The sections were then stained with Hematoxylin and Eosin (H&E) and Periodic Acid–Schiff (PAS) stains. After staining, the sections were dehydrated, treated with xylene, and mounted using DPX. All stained slides were examined under identical lighting conditions. Standard protocols for tissue fixation, processing, and staining were followed as previously described by Bancroft et al. (2019) [49]. Slides were examined using a light microscope.

#### 2.4.6. Immunohistochemical Study

Tissue sections, approximately 5 μm thick, were treated with 3% hydrogen peroxide (H_2_O_2_) for 20 min to block endogenous peroxidase activity, followed by thorough rinsing. The sections were then incubated overnight at 4 °C with primary antibodies, including anti-NF-κB (GTX00763, 1:100, Genetex Co. (Irvine, CA, USA)), anti-Beclin-1 (bs-0056R, 1:200, Bioss Inc. (Woburn, MA, USA)), and anti-Nrf2 (NB100-56565, 1:100, Novus Biologicals (Centennial, CO, USA)). After rinsing with phosphate-buffered saline (PBS), the sections were incubated with an HRP-conjugated EnVision secondary antibody (DAKO) for 20 min, followed by diaminobenzidine (DAB) staining for 10 min. The slides were then washed with PBS, counterstained with hematoxylin, dehydrated, cleared in xylene, and coverslipped. All steps were performed according to standard histological protocols [50].

#### 2.4.7. Morphometric Study

The quantification of the mean area percentage of Beclin-1, NF-κB, and Nrf2 immunoreactivity, as well as PAS staining, was performed by analyzing seven images from seven non-overlapping microscopic fields per group. Image analysis was conducted using Image-Pro Plus software, version 6.0 (Media Cybernetics Inc., Bethesda, MD, USA).

### 2.5. Statistical Analysis

Experimental data were analyzed using IBM SPSS Statistics for Windows, Version 22 (IBM Corp., Armonk, NY, USA). The Shapiro–Wilk test was employed to assess the normality of data distribution. One-way analysis of variance (ANOVA) followed by the post hoc Least Significant Difference (LSD) test was used to determine statistically significant differences between groups in morphometric comparisons. The results are expressed as mean ± standard deviation (SD), with statistical significance set at *p* < 0.05. Groups marked with different letters are considered significantly different (*p* < 0.05).

## 3. Results

### 3.1. Network Pharmacology

#### 3.1.1. Pharmacokinetics, Drug-Likeness, and Physicochemical Property Estimation

The physicochemical properties, pharmacokinetics, and drug-likeness of EA were evaluated using the SwissADME tool. The analysis revealed that EA does not penetrate the blood–brain barrier but demonstrates high gastrointestinal absorption. It fully complies with Lipinski’s Rule of Five, exhibiting no violations. Additionally, EA has a bioavailability score of 0.55 and a molecular weight of 302.19 g/mol—well below the 500 g/mol thresholds—supporting its potential as a promising therapeutic candidate.

#### 3.1.2. Target Collection and Network Construction

**Figure 1:**
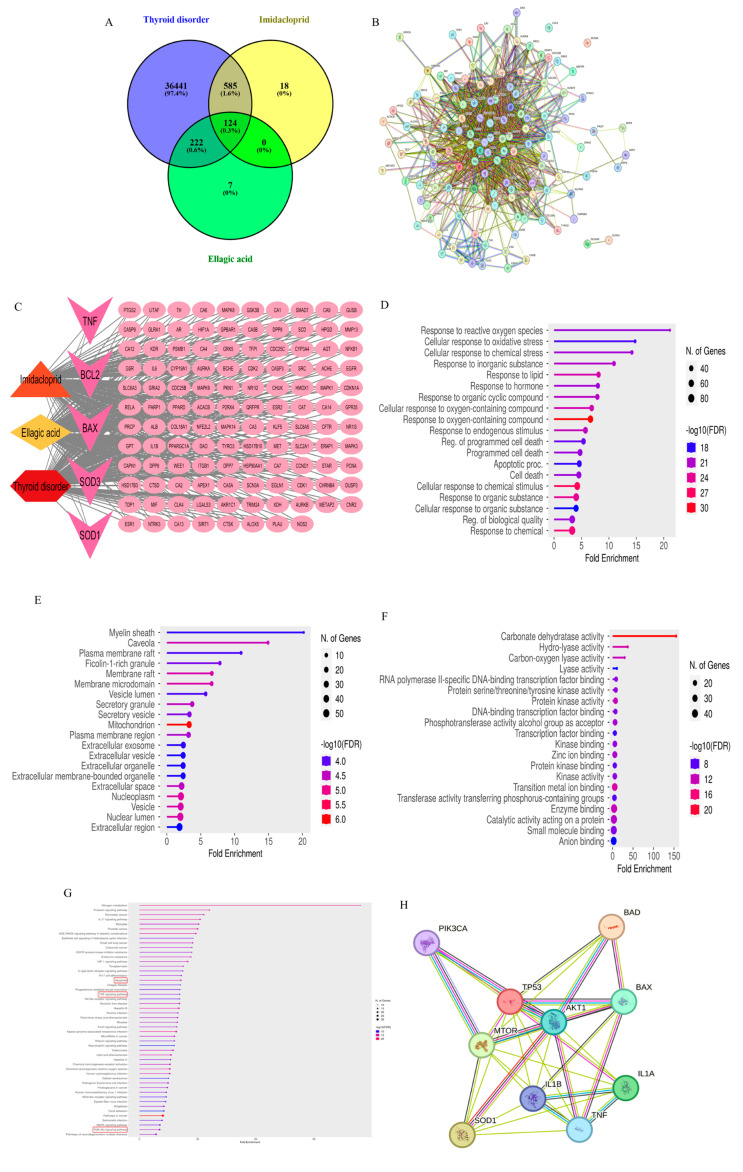
Network pharmacology was constructed revealing (**A**) The 124 intersection targets overlap between the targets of the disease “Thyroid disorder”, drug “Ellagic acid”, insecticide “Imidacloprid”. (**B**) The 124 related targets constructed a PPI network containing 123 nodes and 1251 edges with an average node degree of 20.3. The interaction score was set at 0.4 (medium confidence, which is the default score). (**C**) Disease-Drug-Insecticide-target network, Hexagon: disease, Diamond: drug, Triangle: Insecticide, Ellipse: targets, V-shaped: Experimentally measured targets. Top 20 significantly enriched Gene Ontology (GO) analyses (sorted by Fold Enrichment) (**D**) Biological process (**E**) Cellular component (**F**) Molecular function (**G**) Top 50 KEGG pathways (sorted by Fold Enrichment). The red rectangles indicate the pathways examined in this study. (**H**) Protein-protein interactions between network analysis and in vivo results showing both predicted and experimentally validated interactions.

The Comparative Toxicogenomics Database (CTD) yielded 173 targets for EA, while SwissTargetPrediction and SuperPred identified approximately 100 and 116 targets, respectively. For IMI, 514 targets were obtained from the CTD, 100 from SwissTargetPrediction, 139 from SuperPred, and 12 from the Toxin and Toxin Target Database (T3DB). Regarding thyroid disease-associated targets, CTD, GeneCards (GC), and the Open Targets Platform (OTP) yielded 36,260, 4553, and 9503 targets, respectively. After the removal of duplicates, final validation using the UniProt Knowledgebase identified 353 unique EA targets, 727 IMI targets, and 37,372 thyroid dysfunction-related targets (Supplementary Tables S2–S4). A Venn diagram-based intersection analysis revealed 124 common targets between IMI-induced thyroid dysfunction and EA, suggesting their potential as therapeutic targets (Figure 1A, Supplementary Table S5). STRING analysis identified a protein–protein interaction (PPI) network consisting of 123 nodes and 1251 edges, with an average node degree of 20.3 (Figure 1B). A disease–drug–insecticide–target network was constructed using Cytoscape to visualize and clarify the hub genes (Figure 1C).

GO analysis was conducted across three categories—BPs, CCs, and molecular functions (MFs)—using an FDR cutoff of < 0.05 (Figure 1D–F). KEGG pathway enrichment analysis was performed separately (Figure 1G). Among the top enriched BP terms were GO:1901700 (response to oxygen-containing compounds), GO:0070887 (cellular response to chemical stimulus), GO:0042221 (response to chemical), and GO:0010033 (response to organic substance). The top CC terms included GO:0005739 (mitochondrion), GO:0005654 (nucleoplasm), and GO:0031981 (nuclear lumen). Prominent MF terms included GO:0004089 (carbonate dehydratase activity), GO:0016836 (hydro-lyase activity), GO:0046914 (transition metal ion binding), GO:0019899 (enzyme binding), GO:0016835 (carbon–oxygen lyase activity), and GO:0004672 (protein kinase activity).

A comprehensive KEGG pathway enrichment analysis was performed for the 124 associated targets, using an FDR threshold of < 0.05 to determine statistical significance. From the network analysis results, four key targets—*TNF*, *SOD*, *Bcl*, and *BAX*—were selected for experimental validation, along with their associated pathways: hsa04210 (apoptosis), hsa04151 (*PI3K-Akt* signaling pathway), hsa04668 (*TNF* signaling pathway), hsa04140 (autophagy—animal), and hsa04150 (*mTOR* signaling pathway). These pathways are critically involved in IMI-induced thyroid dysfunction and were investigated as potential mechanisms underlying the protective effects of EA (Figure 1G).

Our analysis revealed that the four selected targets exhibited predicted interactions with several key molecules within these pathways, including *IL-1*, *P53*, *mTOR*, *Akt1*, and *PI3K*. These interactions were further validated experimentally (Figure 1H). Comprehensive details of the GO and KEGG pathway analyses are provided in Supplementary Table S6.

### 3.2. Preparation and Characterization of Ellagic Acid-Loaded Novasomes

**Figure 2:**
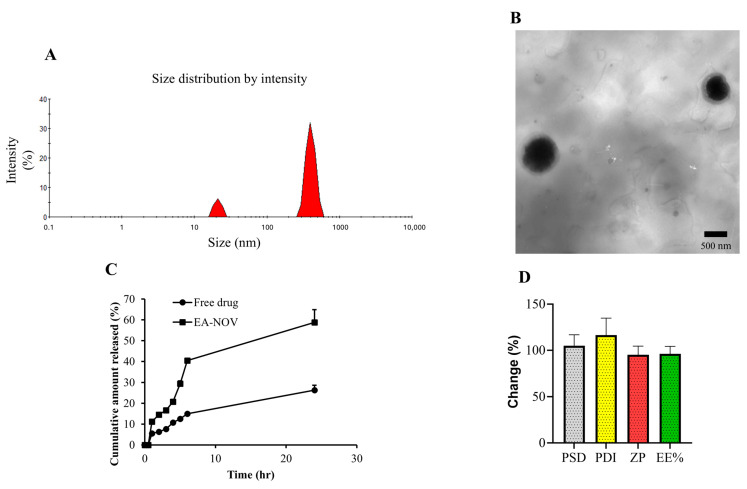
Characterization of EA-NOV prepared using the ethanol injection method. EA-NOV was evaluated for (**A**) particle size, (**B**) morphology by TEM, (**C**) in vitro EA release profile, and (**D**) stability during storage. The ethanol injection technique was successfully applied to formulate EA-NOV. Characterization confirmed spherical morphology, suitable zeta potential, and nanoscale particle size. In vitro release studies demonstrated enhanced EA release from the novasomal formulation compared to free EA. Stability testing revealed that the formulation maintained its physicochemical properties upon storage.

Ellagic acid-loaded novasomes (EA-NOV) were successfully developed using the ethanol injection method. The resulting formulations were characterized by particle size (PS), polydispersity index (PDI), zeta potential (ZP), entrapment efficiency (EE%), TEM, in vitro drug release, and stability studies. Through the use of a Zetasizer Nano, the average particle size of EA-NOV was found to be 501.4 ± 48.63 nm (Figure 2A). The novasomal suspension exhibited uniform particle distribution, as reflected by a PDI of 0.347. The ZP of the system was measured at −49.83 ± 4.81 mV, indicating a suitable surface charge for colloidal stability. TEM imaging confirmed the spherical morphology of the EA-NOV particles, with an average size consistent with the Zetasizer measurements (Figure 2B). The entrapment efficiency of the novasomes was determined to be 85.4 ± 6.08%. In the in vitro release study, the cumulative release profile of EA from the novasomal dispersion was compared with that of free EA (Figure 2C). Free EA showed a release of approximately 14.9% within 6 h and 26.3% over 24 h. In contrast, EA encapsulated in novasomes demonstrated enhanced release, reaching 40.47% and 58.82% at 6 and 24 h, respectively. The stability study showed that the EA-NOV formulations retained their initial physicochemical characteristics over time, with only minor variations observed in the EE%, particle size distribution (PSD), ZP, and PDI (Figure 2D).

Network pharmacology analysis identified 124 overlapping targets among the disease “Thyroid disorder”, the drug “EA”, and the insecticide “IMI” (Figure 1A). These targets were used to construct a protein–protein interaction (PPI) network comprising 123 nodes and 1251 edges, with an average node degree of 20.3, using a medium-confidence interaction score threshold of 0.4 (Figure 1B). A comprehensive disease–drug–insecticide–target network was also generated using Cytoscape, with hexagons representing diseases, diamonds for drugs, triangles for insecticides, ellipses for targets, and V-shapes indicating experimentally validated targets (Figure 1C). GO enrichment analysis revealed the top 20 significantly enriched terms, sorted by fold enrichment, across BPs, CCs, and molecular functions (MFs) (Figure 1D–F). Additionally, the top 50 KEGG pathways were identified based on fold enrichment (Figure 1G). Protein–protein interaction mapping demonstrated convergence between network-predicted targets and in vivo experimental results, highlighting both predicted and experimentally validated interactions (Figure 1H).

### 3.3. Effect of EA-NOV Administration on Serum TSH, Free T3, and Free T4 Levels in IMI-Intoxicated Rats

**Figure 3:**
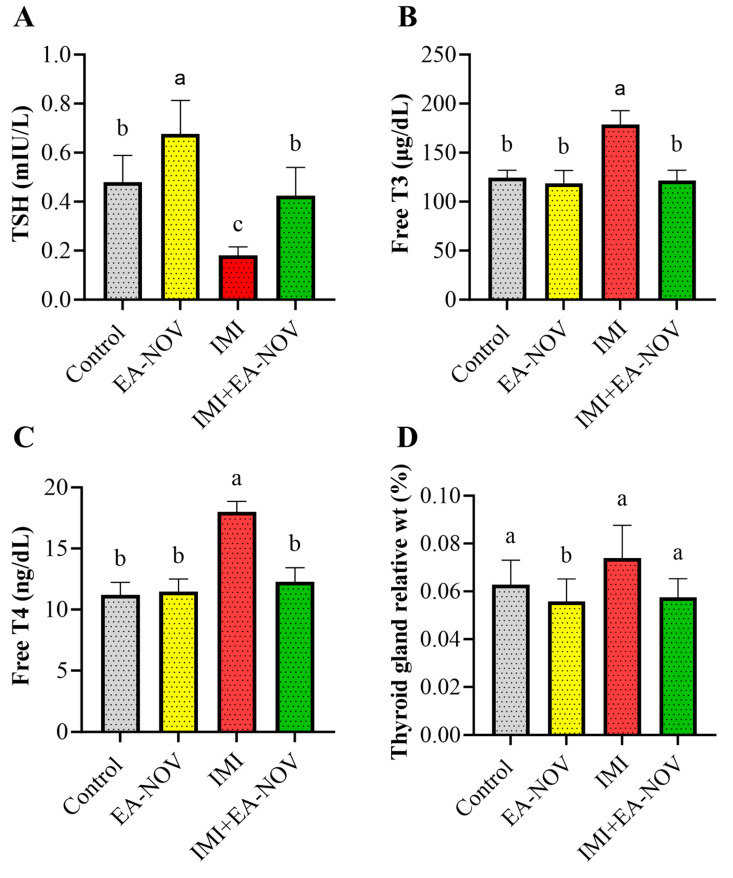
Effects of EA-NOV administration on serum thyroid hormone levels. Serum levels of (**A**) TSH, (**B**) free T3, and (**C**) free T4 were measured in IMI-intoxicated rats. (**D**) thyroid gland relative weight of the different studied groups. Groups labeled with different letters are considered statistically significantly different from one another (*p* < 0.05), including comparisons with the control group. Data are presented as mean ± SD (n = 6).

The effects of EA-NOV administration on serum TSH, free T3, and free T4 levels in IMI-intoxicated rats were evaluated. IMI oral administration significantly decreased serum TSH levels by approximately 62%, while significantly increasing serum free T3 and free T4 levels by 43% and 60%, respectively (Figure 3A–C). The co-administration of EA-NOV with IMI restored serum TSH, free T3, and free T4 levels to near-normal values (Figure 3A–C). Notably, EA-NOV administration alone did not significantly alter TSH, T3, or T4 levels compared to the control group. Additionally, no significant difference in the relative thyroid gland weight was observed between the IMI-treated and IMI+EA-NOV groups (Figure 3D).

### 3.4. Effect of EA-NOV Administration on the Antioxidant Capacity of the Thyroid Gland in IMI-Intoxicated Rats

**Figure 4:**
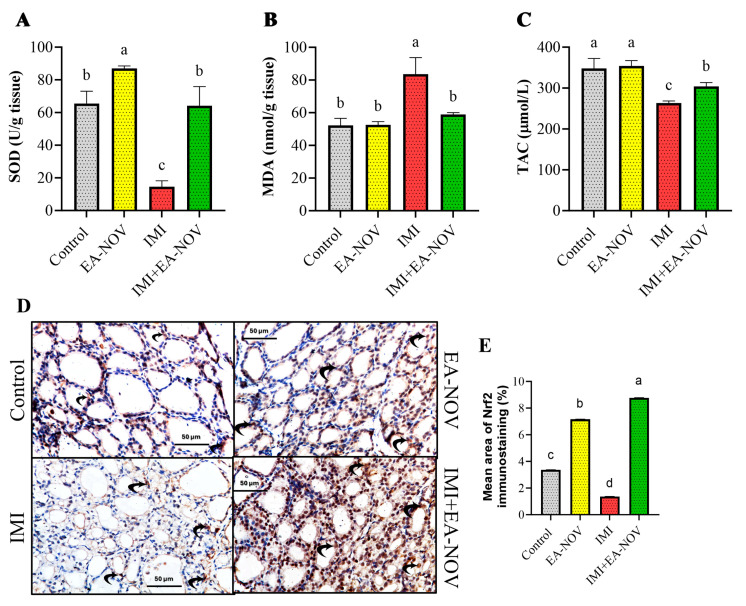
Effect of EA-NOV administration on the antioxidant status of the thyroid gland in IMI-intoxicated rats. The levels of (**A**) superoxide dismutase (SOD), (**B**) malondialdehyde (MDA), and (**C**) total antioxidant capacity (TAC) were measured in thyroid gland tissues across the experimental groups. (**D**) Representative micrographs showing Nrf2 immunostaining in thyroid follicular cells from different experimental groups: control group—mild Nrf2 expression; EA-NOV group—moderate expression; IMI group—weak expression; IMI + EA-NOV group—strong Nrf2 expression. (**E**) Quantitative analysis of the mean area percentage of Nrf2 immunostaining in thyroid follicular cells (Nrf2, ×400 magnification). Groups labeled with different letters are considered statistically significantly different from each other (*p* < 0.05), including comparisons with the control group. Data are presented as mean ± SD (n = 6).

The impact of EA-NOV administration on the antioxidant status of the thyroid gland in IMI-intoxicated rats was also assessed. IMI oral administration significantly reduced the levels of superoxide dismutase (SOD) and total antioxidant capacity (TAC) by 77.8% and 24.3%, respectively, while significantly increasing malondialdehyde (MDA) levels by 60%, in thyroid tissue (Figure 4A–C). In IMI-intoxicated rats, the co-administration of EA-NOV effectively restored SOD, TAC, and MDA levels to near-physiological baselines (Figure 4A–C). EA-NOV administration alone did not significantly affect MDA or TAC levels but resulted in an increase in SOD levels compared to the control.

Immunostaining results for Nrf2 revealed varying expression levels among the experimental groups. Mild Nrf2 positivity was observed in the control group, while moderate positivity was noted in the EA-NOV-treated group. In contrast, the IMI-treated group exhibited weak Nrf2 expression. Notably, combined treatment with IMI and EA-NOV resulted in strong Nrf2 immunostaining, indicating a synergistic effect that significantly enhanced Nrf2 expression (Figure 4D,E).

### 3.5. Effect of EA-NOV Administration on Thyroid Tissue Inflammation Markers in IMI-Intoxicated Rats

**Figure 5:**
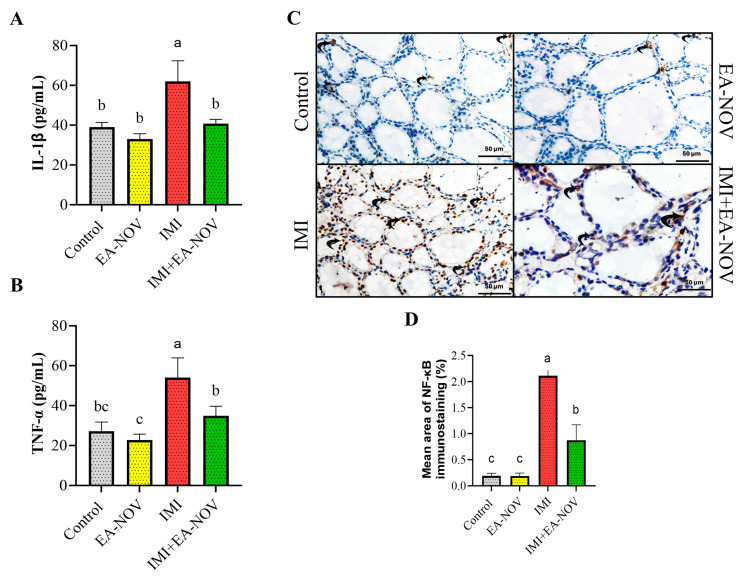
Effect of EA-NOV administration on thyroid tissue inflammation markers in IMI-intoxicated rats. Levels of (**A**) interleukin-1 beta (IL-1β) and (**B**) tumor necrosis factor-alpha (TNF-α) were measured in thyroid tissue homogenates. (**C**) Representative micrographs showing NF-κB immunostaining in thyroid follicular cells from different experimental groups: control group—weak NF-κB expression; EA-NOV group—weak expression; IMI group—strong expression; IMI + EA-NOV group—moderate expression. (**D**) Quantitative analysis of the mean area percentage of NF-κB immunostaining in thyroid follicular cells (NF-κB, ×400 magnification). Groups labeled with different letters are considered statistically significantly different from each other (*p* < 0.05), including comparisons with the control group. Data are presented as mean ± SD (n = 6).

The impact of EA-NOV administration on tissue levels of IL-1β and TNF-α in IMI-intoxicated rats was investigated. IMI oral administration significantly increased IL-1β and TNF-α levels in thyroid tissue by approximately 59% and 99%, respectively (Figure 5A,B). Treatment with EA-NOV in IMI-exposed rats significantly reduced IL-1β and TNF-α levels by 34% and 35%, respectively (Figure 5A,B).

The immunohistochemical analysis of NF-κB expression revealed weakly positive staining in both the control and EA-NOV-treated groups, indicating low basal activation. In contrast, the IMI-treated group exhibited strong NF-κB immunostaining, reflecting pronounced activation. Notably, the IMI + EA-NOV group showed moderately positive NF-κB expression, suggesting that EA-NOV partially attenuates the IMI-induced activation of NF-κB (Figure 5C,D).

### 3.6. Effect of EA-NOV Administration on the Expression of Apoptosis-Related Genes of the Thyroid Gland in IMI-Intoxicated Rats

**Figure 6:**
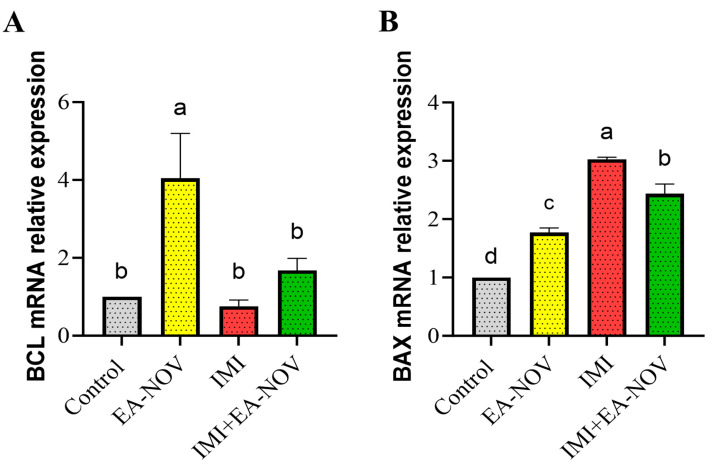
Effect of EA-NOV administration on the mRNA expression of apoptosis-related genes in the thyroid gland of IMI-intoxicated rats. The mRNA expression levels of (**A**) *Bcl-2* and (**B**) *BAX* were measured in thyroid gland tissue homogenates using quantitative real-time PCR (qRT-PCR). Groups denoted by different letters are considered statistically significantly different from each other (*p* < 0.05), including comparisons with the control group. Data are presented as mean ± SD (n = 6).

To assess the apoptotic activity of IMI, real-time PCR was employed to quantify the mRNA expression of *B-cell lymphoma 2 (Bcl-2)* and *Bcl-2-associated X protein (BAX)*. The EA-NOV group exhibited significantly enhanced anti-apoptotic activity, evidenced by a substantial increase in *Bcl-2* mRNA expression compared to the control, IMI, and IMI+EA-NOV groups, as shown in Figure 6A. The transcriptional levels of *BAX*—a marker of cellular apoptosis—in the thyroid tissue of the IMI group were significantly upregulated relative to the control group. However, in the IMI+EA-NOV group, *BAX* mRNA expression was significantly downregulated compared to the IMI group (Figure 6B).

### 3.7. Effect of EA-NOV Administration on the Expression of Autophagy-Related Genes of the Thyroid Gland in IMI-Intoxicated Rats

#### 3.7.1. Autophagy-Inhibitory Mechanisms

**Figure 7:**
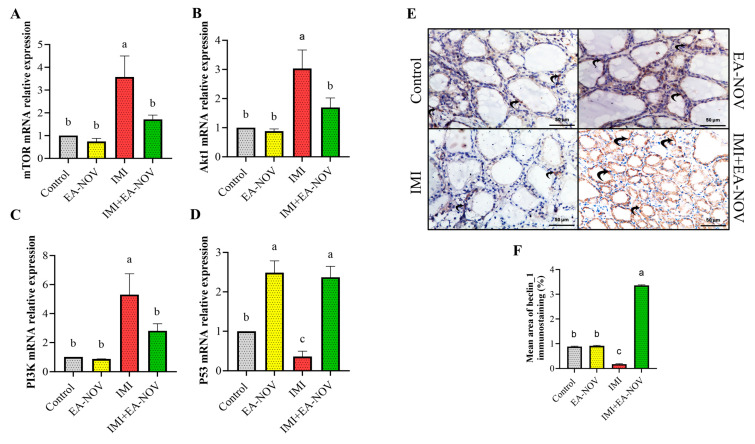
Effect of EA-NOV administration on the mRNA expression of autophagy-related genes in the thyroid gland of IMI-intoxicated rats. The mRNA expression levels of (**A**) *mTOR*, (**B**) *Akt1*, (**C**) *PI3K*, and (**D**) *p53* were measured in thyroid gland tissue homogenates using qRT-PCR. (**E**) Representative micrographs of thyroid gland sections from different experimental groups showing *Beclin-1* immunostaining: the control group exhibited mild *Beclin-1* staining in follicular cells; the EA-NOV group showed a similar mild staining pattern; the IMI group displayed weak *Beclin-1* staining; and the IMI+EA-NOV group demonstrated strong *Beclin-1* immunostaining in follicular cells. (**F**) Quantitative analysis of the mean area percentage of *Beclin-1* immunostaining in follicular cells (Beclin-1 ×400). Groups labeled with different letters are considered statistically significantly different from each other (*p* < 0.05), including comparisons with the control group. Data are presented as mean ± SD (n = 6).

One of the primary pathways regulating autophagy is the well-established *PI3K/Akt/mTOR* signaling cascade [51]. To elucidate the mechanisms by which IMI influences thyroid autophagy, its effect on the mRNA expression levels of *mammalian target of rapamycin* (*mTOR*, Figure 7A), *protein kinase B* (*Akt*, Figure 7B), and *phosphoinositide 3-kinase* (*PI3K*, Figure 7C) was examined. The results revealed that the expression levels of *mTOR*, *Akt*, and *PI3K* were significantly elevated following IMI administration, indicating increased activity of the *PI3K/Akt/mTOR* signaling pathway in the thyroid tissue of IMI-treated rats. However, the co-administration of EA-NOV with IMI effectively restored the mRNA expression of *mTOR*, *Akt*, and *PI3K* to near-normal levels in the thyroid gland (Figure 7A–C).

#### 3.7.2. Autophagy-Induction Mechanisms

The mRNA expression level of *tumor protein p53 (p53)* was significantly reduced in the IMI group compared to the control group. Conversely, *p53* mRNA expression was notably elevated in the IMI+EA-NOV group compared to the IMI group (Figure 7D). Beclin-1 protein expression was mildly positive in both the control and EA-NOV groups, indicating baseline autophagic activity. In the IMI-treated group, *Beclin-1* expression was weakly positive, suggesting diminished autophagy. However, in the IMI+EA-NOV group, *Beclin-1* expression was strongly positive, reflecting a marked upregulation of autophagy. These findings suggest that EA-NOV mitigates the IMI-induced suppression of autophagy, potentially enhancing cellular protection (Figure 7E,F).

### 3.8. Histological Findings

**Figure 8:**
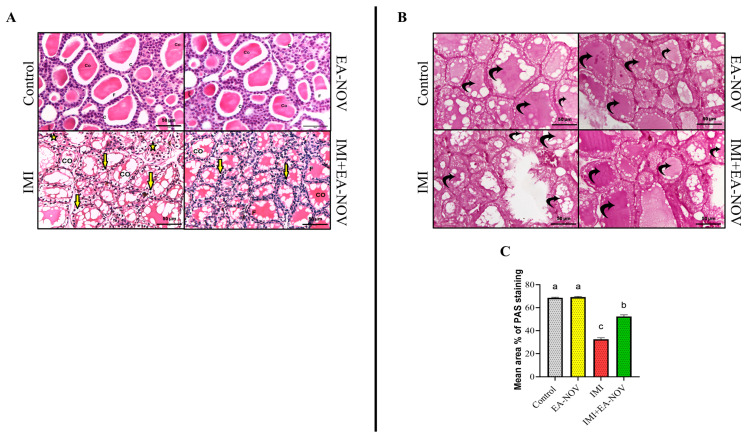
Effect of EA-NOV administration on the histological and histochemical features of the thyroid gland in IMI-intoxicated rats. (**A**) H&E-stained sections show normal thyroid follicles in the control group, lined with simple cuboidal epithelium and filled with a homogeneous acidophilic colloid (Co). The EA-NOV group exhibited similar histological architecture. The IMI group showed vacuolation in the follicular epithelium (yellow arrow), disorganized follicles (yellow star), and a highly vacuolated colloid. In contrast, the IMI+EA-NOV group displayed improved follicular structure with fewer vacuolations in both the follicular cells (black arrow) and colloid. (**B**) PAS-stained sections revealed strong positive colloid staining in the control and EA-NOV groups, a marked reduction in the IMI group, and a moderate reaction in the IMI+EA-NOV group. (**C**) Mean area percentage of PAS staining in the follicular colloid. Groups labeled with different letters are considered statistically significantly different (*p* < 0.05). Data are presented as mean ± SD (n = 6).

In both the control and EA-NOV groups, H&E-stained thyroid sections exhibited normal follicular architecture, characterized by cuboidal follicular cells with centrally located, round nuclei. The follicular lumina contained a uniformly acidophilic colloid (Figure 8A, control and EA-NOV). In contrast, the IMI group showed marked histological alterations, including the widespread cytoplasmic vacuolation of the follicular cells, disrupted follicular organization, and irregular follicle morphology. Additionally, some follicles contained a highly vacuolated colloid (Figure 8A, IMI). In the IMI+EA-NOV group, thyroid sections revealed an almost normal architecture, with reduced vacuolation in both the follicular cells and the colloid, indicating a protective effect of EA-NOV against IMI-induced histopathological changes (Figure 8A, IMI+EA-NOV).

PAS histochemical analysis revealed a strong positive reaction in the follicular colloid of thyroid sections from both the control (Figure 8B, control) and EA-NOV-treated groups (Figure 8B, EA-NOV). In contrast, the IMI-treated group showed a mild positive reaction in the colloid (Figure 8B, IMI), indicating a reduction in glycoprotein content. The IMI+EA-NOV group exhibited a moderate PAS reaction in the follicular colloid (Figure 8B, IMI+EA-NOV), suggesting partial restoration. The quantitative analysis of the mean area percentages of PAS staining for all groups is shown in Figure 8C. A significant decrease in PAS reactivity was observed in the IMI group compared to the control group, whereas the IMI+EA-NOV group demonstrated a significant increase in PAS expression compared to the IMI group.

## 4. Discussion

THs play a crucial role as homeostatic regulators of metabolism and developmental processes [52]. Oxidative stress and inflammatory cascades are key contributors to thyroid dysfunction. Globally, exposure to environmental pollutants remains a significant health risk, particularly in low-income countries [53]. Neonicotinoids have a profound impact on the endocrine system, primarily through their toxic effects and disruption of the hypothalamic–pituitary–thyroid (HPT) axis in rats [52]. IMI, a widely used neonicotinoid pesticide, has been shown to exacerbate damage across various physiological tissues, with some effects potentially leading to life-threatening outcomes [53,54]. EA, a natural polyphenol found in various fruits, has been widely recognized for its potent anti-inflammatory, anti-apoptotic, and antioxidant properties [55]. To the best of our knowledge, this is the first study to report the protective effect of EA against IMI-induced oxidative, inflammatory, and apoptotic damage in the rat thyroid, highlighting its potential protective role through the modulation of the autophagy pathway.

Currently, network pharmacology serves as a powerful analytical framework for elucidating the molecular mechanisms underlying drug–disease interactions. In the present study, we employed systems pharmacology and toxicological approaches to explore the potential molecular mechanisms of EA in mitigating IMI-induced thyroid dysfunction. A total of 124 common targets were identified as potential mediators of EA’s protective effects against IMI-induced thyroid impairment. However, considering the limitations of network pharmacology—such as the lack of sufficient clinical data, which may affect its applicability and reliability [56]—experimental validation was conducted to support and confirm the computational findings.

The available evidence supports a critical role of oxidative stress in the pathogenesis of such conditions. Elevated ROS levels following IMI exposure may lead to cellular damage and death, including oxidative injury to proteins, lipids, and DNA, thereby contributing to the deterioration of thyroid gland structure and function [57]. This study evaluated total antioxidant capacity (TAC) and the antioxidant activity of enzyme superoxide dismutase (SOD), along with malondialdehyde (MDA) as a biomarker of lipid peroxidation. IMI exposure resulted in a significant increase in MDA levels in the thyroid, accompanied by a marked decrease in both SOD activity and TAC. These findings indicate pronounced oxidative stress in the thyroid following IMI administration. Consistent with our results, Zhou et al. reported a similarly dysregulated redox status in tissues after IMI exposure. Elevated levels of lipid peroxidation and protein oxidation are key contributors to the oxidative damage induced by IMI [58]. It is important to emphasize that thyroid cells are particularly vulnerable to oxidative stress following IMI exposure, which leads to the excessive generation of endogenous ROS. This heightened oxidative burden can result in mitochondrial dysfunction, irreversible cellular damage, apoptosis, and impaired antioxidant defense mechanisms [6]. Our histopathological analysis revealed notable vacuolation in both the follicular cells and colloid, along with the presence of deformed follicles. Additionally, a weak PAS reaction was observed in the colloid, suggesting structural damage to the gland and a consequent decline in thyroid gland activity. Interestingly, despite the observed histological damage, there was no corresponding decrease in serum T3 and T4 levels, as might have been expected. This discrepancy may be attributed to the subchronic nature of IMI exposure in our model, as opposed to chronic administration. It is plausible that oxidative damage to the follicular cell membranes led to the increased leakage of T4 into the circulation. The elevated plasma T3 levels observed in the IMI group may also result from the enhanced peripheral conversion of T4 to T3 in epithelial cells. Furthermore, the increased levels of T3 and T4 likely exert negative feedback on the pituitary gland, resulting in suppressed TSH secretion [52]. Consistent with our findings, Ibrahim et al. and Pandey et al. reported that IMI disrupts the pituitary–thyroid axis in both neonatal rats and wild bird species [52,59]. 

EA is a well-known antioxidant that effectively counteracts the harmful effects of ROS [60]. Our findings are supported by previous studies demonstrating that EA effectively restores serum T3, T4, and TSH to normal levels [61]. EA prevents IMI-induced toxicity and ROS-mediated damage to the cell membrane by shielding follicular cells from oxidative stress. To further investigate the antioxidant properties of EA, the expression of Nrf2 was assessed. Nrf2 is a key transcription factor that safeguards cells against oxidative injury by upregulating the expression of antioxidant genes [62]. According to our research, EA pretreatment enhanced the expression of the Nrf2 protein, thereby improving resistance to oxidative stress. Our findings are consistent with those of Al-Tamimi et al. and Wang et al., who demonstrated that EA treatment significantly upregulated Nrf2 expression, thereby preventing Parkinson’s disease and streptozotocin (STZ)-induced diabetic nephropathy in rats, respectively [63,64]. Wei et al. similarly demonstrated that EA protects dopamine-releasing neurons against rotenone-induced neurotoxicity through the Nrf2 signaling pathway [65]. 

There is a close interaction between pro-inflammatory processes and oxidative stress [62]. The inflammatory response observed in thyroid tissue following IMI exposure was marked by an upregulation of NF-κB immunohistochemical expression, which in turn promoted the secretion of pro-inflammatory cytokines, including IL-1β and TNF-α. This response can be attributed to oxidative stress-induced cellular damage, mediated through cytoskeletal protein degradation and the lipid peroxidation of membrane phospholipids. These oxidative insults may activate pro-inflammatory signaling pathways, thereby initiating a cascade of inflammatory events. [66]. On the other hand, the anti-inflammatory properties of EA stem from its ability to inhibit NF-κB activation, suggesting its potential as a thyroid-protective agent. EA may also play a significant role in regulating key physiological mechanisms involved in inflammation and protein degradation [55]. EA can inhibit the activation of TNF-α-dependent NF-κB, as well as other pro-inflammatory cytokines and chemokines, such as IL-1β. Our findings are supported by previous studies demonstrating EA’s anti-inflammatory effects in cardiac fibrosis [14], as well as in the liver and brain of rats treated with D-galactose [67].

As previously reported, an imbalance in the redox system and inflammatory processes can lead to oxidative DNA damage and aberrant protein expression, ultimately promoting apoptosis [58]. Consequently, the suppression of oxidative damage and inflammation is crucial for preventing programmed cell death. The findings of this study revealed that IMI administration in rats induced apoptosis in the thyroid gland, as evidenced by the significant increase in the pro-apoptotic marker *Bax* and the simultaneous decrease in the anti-apoptotic marker *Bcl* within thyroid tissue. These observations align with previous research demonstrating that IMI exposure activates apoptotic pathways in both thyroid and brain tissues through the upregulation of apoptotic markers, thereby reinforcing the validity of our results [59,68]. Conversely, in the current study, EA administration significantly decreased Bax expression and increased Bcl levels, likely due to its ability to attenuate oxidative stress and inflammation. This was associated with a marked reduction in thyroid gland apoptosis. Our findings are consistent with previous studies demonstrating the anti-apoptotic effects of EA, which was shown to ameliorate apoptosis in pancreatic beta cells in type 2 diabetes [69], in a busulfan-induced rat model of testicular damage [70], and in amikacin-induced nephrotoxicity [71] by downregulating *Bax* and upregulating *Bcl* expression. 

Mechanistically, autophagy plays a pivotal role in the pathophysiology of IMI-induced toxicity [72]. Intracellular autophagy is a self-destructive process that occurs in most stressed cells to maintain cellular homeostasis, preserve genomic stability, and support metabolic adaptation by degrading and recycling damaged organelles and CCs [10]. Autophagy is essential for cellular adaptation to conditions such as endoplasmic reticulum (ER) stress, hypoxia, and oxidative stress, and it also plays a key role in regulating the cell cycle and apoptosis [73].

Notably, the *phosphoinositide 3-kinase (PI3K)* signaling pathway serves as a key regulatory mechanism in the modulation of autophagy [74]. Furthermore, *Akt* acts as a downstream effector of *PI3K*. The conventional role of the *PI3K/Akt* signaling pathway is to exert an inhibitory effect on autophagy, primarily through the activation of *mTOR* [75]. *mTOR* inhibits the early stages of autophagy by suppressing autophagy-activating genes and directly blocking the initiation of autophagosome formation [76].

Our data revealed that IMI treatment significantly upregulated the mRNA expression of *PI3K* and *Akt*. Consistent with our findings, Xie et al. demonstrated that IMI exposure induces lung injury in mice by activating the *PI3K/Akt/NF-κB* signaling pathway [72]. On the other hand, EA co-treatment with IMI restored *PI3K* expression to normal levels, consistent with the findings of Wang et al., who reported that EA reduced PI3K expression in endometrial cancer [77]. In addition, Yu et al. demonstrated that EA treatment suppresses the *PI3K/Akt* signaling pathway [78]. In addition, EA administration restored normal mRNA expression levels of mTOR in thyroid gland tissues. This finding is supported by Xiaohe Li et al., who reported that EA treatment decreased *mTOR* phosphorylation levels [79]. These results suggest that EA induces autophagy by inhibiting the *PI3K/Akt/mTOR* signaling pathway in the thyroid gland.

There is a significant relationship between autophagy and *p53*. Cytoplasmic *p53* can act within mitochondria to promote cell death and simultaneously activate autophagy. This occurs through the enhancement of autophagy-related gene transcription by *p53*. Specifically, p53 initiates autophagy by suppressing the *mTOR* pathway, which normally inhibits autophagy, and/or by upregulating key autophagy-related genes, including *Unc-51-like kinase 1 (ULK1)*, *autophagy-related gene 7 (Atg7)*, *and damage-regulated autophagy modulator (DRAM)*, a lysosomal protein that facilitates the formation of autophagic vacuoles [80]. In addition, *p53* enhances the expression of Beclin-1, a key regulator of autophagy initiation [81]. Beclin-1 was the first autophagy-related gene identified in mammals and is a crucial component in the initiation and formation of autophagosomes [82]. Furthermore, *Beclin-1* regulates the activity of *PI3K* and promotes the activation of LC3, facilitating autophagosome formation [83]. Our results showed a significant increase in *p53* mRNA expression levels and enhanced immunohistochemical staining following EA supplementation. These findings are consistent with those of Baeeri et al., who demonstrated that EA increased *p53* expression in phosalone-induced senescence in rat embryonic fibroblasts [84], and Cai et al., who reported that EA attenuates hypoxic–ischemic brain injury by modulating autophagy through the upregulation of *Beclin-1* levels [85].

## 5. Conclusions

In conclusion, EA appears to exert antioxidant, anti-inflammatory, and anti-apoptotic effects. Notably, it also promotes autophagy by inhibiting the *PI3K/Akt/mTOR* pathway and activating the *p53/Beclin-1* signaling pathway, thereby mitigating thyroid dysfunction and tissue damage. However, further clinical studies are necessary to validate these molecular targets and to clarify the potential therapeutic role of EA in patients with thyroid dysfunction.

## Figures and Tables

**Table 1:** A list of primer sequences used for RT-PCR.

Target Gene	Primer Sequences	Accession Number	Amplicon Length (bp)
*B-actin*	CGGTCAGGTCATCACTATCG	NM_031144.3	79
	TAGTTTCATGGATGCCACAG		
*PI3K*	CGTATCCACCTGTCCTCTCC	NM_001371300.3	199
	CTCCTTCCAAGCCTCAGTGA		
*Akt1*	CTGCCCTTCTACAACCAGGA	NM_033230.3	177
	GTGCTGCATGATCTCCTTGG		
*mTOR*	TCTGCACTTGTTGTTGCCT	NM_019906.2	150
	ACAATCGGGTGAATGATGCG		
*BAX*	CAGCTCTGAACAGATCAT	NM_017059.2	218
	AGTCTGTATCCACATCAG		
*Bcl*	AGGGATGGAGGAGGAGCTTA	NM_022698.2	175
	TTGTCGCATCTGTGTTGC		
*P53*	GGCTCCGACTATACCACTATCCAC	U07020.1	129
	GTCCCGTCCCAGAAGATTC		

## Data Availability

The raw data supporting the conclusions of this article will be made available by the authors on request.

## Supplementary Materials

The following supporting information can be downloaded at https://www.mdpi.com/article/10.3390/toxics13050355/s1. Table S1 presents the chemical structures of EA and IMI in SMILES format, obtained from PubChem. Table S2 lists 37,372 common disease-related targets identified from three databases. Table S3 includes 353 targets for EA retrieved from three databases after removing duplicates, while Table S4 shows 727 targets for IMI obtained from four databases after duplicate removal. Table S5 highlights 124 overlapping targets between EA and IMI. Finally, Table S6 provides the results of enrichment analysis of the common targets.

## References

[1] Vohra P., Khera K. (2015). A Three Generation Study with Effect of Imidacloprid in Rats: Biochemical and Histopathological Investigation. Toxicol. Int..

[2] Wang Y., Xu P., Chang J., Li W., Yang L., Tian H. (2020). Unraveling the toxic effects of neonicotinoid insecticides on the thyroid endocrine system of lizards. Environ. Pollut..

[3] Zhao G.-P., Li J.-W., Yang F.-W., Yin X.-F., Ren F.-Z., Fang B., Pang G.-F. (2021). Spermiogenesis toxicity of imidacloprid in rats, possible role of CYP3A4. Chemosphere.

[4] Yi X., Zhang C., Liu H., Wu R., Tian D., Ruan J., Zhang T., Huang M., Ying G. (2019). Occurrence and distribution of neonicotinoid insecticides in surface water and sediment of the Guangzhou section of the Pearl River, South China. Environ. Pollut..

[5] Hernández A.F., Bennekou S.H., Hart A., Mohimont L., Wolterink G. (2020). Mechanisms underlying disruptive effects of pesticides on the thyroid function. Curr. Opin. Toxicol..

[6] Kochman J., Jakubczyk K., Bargiel P., Janda-Milczarek K. (2021). The Influence of Oxidative Stress on Thyroid Diseases. Antioxidants.

[7] Panda S., Kar A., Singh M., Singh R.K., Ganeshpurkar A. (2021). Syringic acid, a novel thyroid hormone receptor-β agonist, ameliorates propylthiouracil-induced thyroid toxicity in rats. J. Biochem. Mol. Toxicol..

[8] Miao Z., Miao Z., Wang S., Wu H., Xu S. (2022). Exposure to imidacloprid induce oxidative stress, mitochondrial dysfunction, inflammation, apoptosis and mitophagy via NF-kappaB/JNK pathway in grass carp hepatocytes. Fish. Shellfish. Immunol..

[9] Qumsani A.T. (2025). Exploring the Effects of Imidacloprid on Liver Health and the Microbiome in Rats: A Comprehensive Study. Microorganisms.

[10] Chen D., Huang X., Lu S., Deng H., Gan H., Huang R., Zhang B. (2019). miRNA-125a modulates autophagy of thyroiditis through PI3K/Akt/mTOR signaling pathway. Exp. Ther. Med..

[11] Zuo X., Wei X., Ju C., Wang X., Zhang Z., Ma Y., Zhu Z., Li X., Song Z., Luo L. (2022). Protective Effect of Photobiomodulation against Hydrogen Peroxide-Induced Oxidative Damage by Promoting Autophagy through Inhibition of PI3K/AKT/mTOR Pathway in MC3T3-E1 Cells. Oxid. Med. Cell Longev..

[12] Liu X., Zhou C., Cheng B., Xiong Y., Zhou Q., Wan E., He Y. (2024). Genipin promotes the apoptosis and autophagy of neuroblastoma cells by suppressing the PI3K/AKT/mTOR pathway. Sci. Rep..

[13] Huang P.-Y., Hsieh Y.-H., Ting Y.-H., Lee C.-C., Tsai J.-P. (2024). Ellagic acid ameliorates renal fibrogenesis by blocking epithelial-to-mesenchymal transition. Tzu Chi Med. J..

[14] Mannino F., Imbesi C., Bitto A., Minutoli L., Squadrito F., D’Angelo T., Booz C., Pallio G., Irrera N. (2023). Anti-oxidant and anti-inflammatory effects of ellagic and punicic acid in an in vitro model of cardiac fibrosis. Biomed. Pharmacother..

[15] Mohammadinejad A., Mohajeri T., Aleyaghoob G., Heidarian F., Oskuee R.K. (2022). Ellagic acid as a potent anticancer drug: A comprehensive review on in vitro, in vivo, in silico, and drug delivery studies. Biotechnol. Appl. Biochem..

[16] Ramadan W.S., Alkarim S., Moulay M., Alrefeai G., Alkudsy F., Hakeem K.R., Iskander A. (2023). Modulation of the Tumor Microenvironment by Ellagic Acid in Rat Model for Hepatocellular Carcinoma: A Potential Target against Hepatic Cancer Stem Cells. Cancers.

[17] Aslan A., Gok O., Beyaz S., Arslan E., Erman O., Ağca C.A. (2020). The preventive effect of ellagic acid on brain damage in rats via regulating of Nrf-2, NF-kB and apoptotic pathway. J. Food Biochem..

[18] Warpe V.S., Mali V.R., Bodhankar S.L., Mahadik K.R. (2015). Cardioprotective effect of ellagic acid on doxorubicin induced cardiotoxicity in wistar rats. J. Acute Med..

[19] Shabani M., Bayrami D., Moghadam A.A., Jamali Z., Salimi A. (2023). Pretreatment of ellagic acid protects ifosfamide-induced acute nephrotoxicity in rat kidneys: A mitochondrial, histopathological and oxidative stress approaches. Toxicol. Rep..

[20] Chen S., Zhou M., Ying X., Zhou C. (2021). Ellagic acid protects rats from chronic renal failure via MiR-182/FOXO3a axis. Mol. Immunol..

[21] Gramec Skledar D., Tomašič T., Dolenc M.S., Mašič L.P., Zega A. (2019). Evaluation of endocrine activities of ellagic acid and urolithins using reporter gene assays. Chemosphere.

[22] Di Dalmazi G., Giuliani C. (2021). Plant constituents and thyroid: A revision of the main phytochemicals that interfere with thyroid function. Food Chem. Toxicol..

[23] Mahmood S.M., Measer A.A. (2022). Effect of Hyperthyroidism on Lipid Metabolism and Evaluation of the Protective Role of Pomegranate Juice against the Risk of Oxidative Stress in Albino Rats. Int. J. Drug Deliv. Technol..

[24] González-Sarrías A., García-Villalba R., Núñez-Sánchez M.Á., Tomé-Carneiro J., Zafrilla P., Mulero J., Tomás-Barberán F.A., Espín J.C. (2015). Identifying the limits for ellagic acid bioavailability: A crossover pharmacokinetic study in healthy volunteers after consumption of pomegranate extracts. J. Funct. Foods.

[25] Darwish A.B., Salama A., Al-Samadi I.E.I. (2025). Formulation, optimisation, and evaluation of Lornoxicam-loaded Novasomes for targeted ulcerative colitis therapy: In vitro and in vivo investigations. J. Drug Target..

[26] National Center for Biotechnology Information PubChem Compound Summary for CID 5281855, E.A. https://pubchem.ncbi.nlm.nih.gov/compound/Ellagic-Acid.

[27] National Center for Biotechnology Information PubChem Compound Summary for CID 86287518, I.P. https://pubchem.ncbi.nlm.nih.gov/compound/Imidacloprid.

[28] Daina A., Michielin O., Zoete V. (2017). SwissADME: A free web tool to evaluate pharmacokinetics, drug-likeness and medicinal chemistry friendliness of small molecules. Sci. Rep..

[29] Davis A.P., Wiegers T.C., Johnson R.J., Sciaky D., Wiegers J., Carolyn J. (2023). Mattingly Comparative Toxicogenomics Database (CTD): Update 2023. Nucleic Acids Res..

[30] Daina A., Michielin O., Zoete V. (2019). SwissTargetPrediction: Updated data and new features for efficient prediction of protein targets of small molecules. Nucleic Acids Res..

[31] Nickel J., Gohlke B.-O., Erehman J., Banerjee P., Rong W.W., Goede A., Dunkel M., Preissner R. (2014). SuperPred: Update on drug classification and target prediction. Nucleic Acids Res..

[32] Wishart D., Arndt D., Pon A., Sajed T., Guo A.C., Djoumbou Y., Knox C., Wilson M., Liang Y., Grant J. (2015). T3DB: The toxic exposome database. Nucleic Acids Res..

[33] Stelzer G., Rosen N., Plaschkes I., Zimmerman S., Twik M., Fishilevich S., Stein T.I., Nudel R., Lieder I., Mazor Y. (2016). The GeneCards Suite: From Gene Data Mining to Disease Genome Sequence Analyses. Curr. Protoc. Bioinform..

[34] Ochoa D., Hercules A., Carmona M., Suveges D., Baker J., Malangone C., Lopez I., Miranda A., Cruz-Castillo C., Fumis L. (2023). McDonagh The next-generation Open Targets Platform: Reimagined, redesigned, rebuilt. Nucleic Acids Res..

[35] The UniProt C. (2023). UniProt: The Universal Protein Knowledgebase in 2023. Nucleic Acids Res..

[36] Bardou P., Mariette J., Escudié F., Djemiel C., Klopp C. (2014). jvenn: An interactive Venn diagram viewer. BMC Bioinform..

[37] Szklarczyk D., Kirsch R., Koutrouli M., Nastou K., Mehryary F., Hachilif R., Gable A.L., Fang T., Doncheva N.T., Pyysalo S. (2023). The STRING database in 2023: Protein–protein association networks and functional enrichment analyses for any sequenced genome of interest. Nucleic Acids Res..

[38] Shannon P., Markiel A., Ozier O., Baliga N.S., Wang J.T., Ramage D., Amin N., Schwikowski B., Ideker T.J.G.R. (2003). Cytoscape: A software environment for integrated models of biomolecular interaction networks. Genome Res..

[39] Ge S.X., Jung D., Yao R. (2020). ShinyGO: A graphical gene-set enrichment tool for animals and plants. Bioinformatics.

[40] Abd El-Emam M.M., Behairy A., Mostafa M., Khamis T., Osman N.M.S., Alsemeh A.E., Mansour M.F. (2024). Chrysin-loaded PEGylated liposomes protect against alloxan-induced diabetic neuropathy in rats: The interplay between endoplasmic reticulum stress and autophagy. Biol. Res..

[41] Mosallam S., Ragaie M.H., Moftah N.H., Elshafeey A.H., Abdelbary A.A. (2021). Use of Novasomes as a Vesicular Carrier for Improving the Topical Delivery of Terconazole: In Vitro Characterization, In Vivo Assessment and Exploratory Clinical Experimentation. Int. J. Nanomed..

[42] Cornélio Favarin D., Teixeira M.M., de Andrade E.L., de Freitas Alves C., Chica J.E.L., Sorgi C.A., Faccioli L.H., Paula A.P. (2013). Anti-Inflammatory Effects of Ellagic Acid on Acute Lung Injury Induced by Acid in Mice. Mediat. Inflamm..

[43] Aishwarya V., Solaipriya S., Sivaramakrishnan V. (2021). Role of ellagic acid for the prevention and treatment of liver diseases. Phytother. Res..

[44] WHO (2010). The WHO Recommended Classification of Pesticides by Hazard and Guidelines to Classification 2009.

[45] Mikolić A., Karačonji I.B. (2018). Imidacloprid as reproductive toxicant and endocrine disruptor: Investigations in laboratory animals. Arch. Ind. Hyg. Toxicol..

[46] Tomlin C.D.S. (2006). The pesticides manual: A world compendium. Br. Crop Prot. Counc..

[47] Pardridge W.M., Mietus L.J. (1980). Influx of Thyroid Hormones into Rat Liver In Vivo: Differential Availability of Thyroxine and Triiodothyronine Bound by Plasma Proteins. J. Clin. Investig..

[48] Yuan J.S., Reed A., Chen F., Stewart C.N. (2006). Statistical analysis of real-time PCR data. BMC Bioinform..

[49] Spencer L.T., Suvarna S.K., Layton C., Bancroft J.D. (2019). 7—Microtomy for paraffin and frozen sections. Bancroft’s Theory and Practice of Histological Techniques.

[50] Chaplin A.J. (1985). Manual of histological techniques. J. D. Bancroft and H. C. Cook. Churchill Livingstone, Edinburgh, London, Melbourne, New York, 1984. No. of pages: Ii + 274. Price: £12.50. ISBN: 0 443 02870 2. J. Pathol..

[51] Liu K., Yang Y., Zhou F., Xiao Y., Shi L. (2020). Inhibition of PI3K/AKT/mTOR signaling pathway promotes autophagy and relieves hyperalgesia in diabetic rats. NeuroReport.

[52] Pandey S.P., Mohanty B. (2015). The neonicotinoid pesticide imidacloprid and the dithiocarbamate fungicide mancozeb disrupt the pituitary–thyroid axis of a wildlife bird. Chemosphere.

[53] El Gazzar W.B., Bayoumi H., Youssef H.S., Ibrahim T.A., Abdelfatah R.M., Gamil N.M., Iskandar M.K., Abdel-Kareim A.M., Abdelrahman S.M., Gebba M.A. (2024). Role of IRE1α/XBP1/CHOP/NLRP3 Signalling Pathway in Neonicotinoid Imidacloprid-Induced Pancreatic Dysfunction in Rats and Antagonism of Lycopene: In Vivo and Molecular Docking Simulation Approaches. Toxics.

[54] Samala S., Mekala L., Doppalapudi M., Alla G.R., Rao R. (2023). Evaluation of Imidacloprid Induced Changes in Thyroid Gland and its Amelioration with Withania somnifera in Wistar Rats. J. Vet. Sci. Biotechnol..

[55] Gupta A., Kumar R., Ganguly R., Singh A.K., Rana H.K., Pandey A.K. (2021). Antioxidant, anti-inflammatory and hepatoprotective activities of Terminalia bellirica and its bioactive component ellagic acid against diclofenac induced oxidative stress and hepatotoxicity. Toxicol. Rep..

[56] Liu Z., Huang H., Yu Y., Li L., Shi X., Wang F. (2023). Exploring the mechanism of ellagic acid against gastric cancer based on bioinformatics analysis and network pharmacology. J. Cell Mol. Med..

[57] Vieira C.E.D., Pérez M.R., Acayaba R.D.A., Raimundo C.C.M., Martinez C.B.D.R. (2018). DNA damage and oxidative stress induced by imidacloprid exposure in different tissues of the Neotropical fish *Prochilodus lineatus*. Chemosphere.

[58] Farag A.A., Bayoumi H., Radwaan S.E., El Gazzar W.B., Youssef H.S., Nasr H.E., Badr A.M., Mansour H.M., Elalfy A., Sayed A.E.-D.H. (2024). Melatonin counteracts polyethylene microplastics induced adreno-cortical damage in male albino rats. Ecotoxicol. Environ. Saf..

[59] Ibrahim K., El-Desouky M., Abou-Yousef H., Gabrowny K., El-Sayed A.J. (2015). Imidacloprid and/or esfenvalerate induce apoptosis and disrupt thyroid hormones in neonatal rats. Glob. J. Biotechnol. Biochem..

[60] Alfei S., Marengo B., Zuccari G. (2020). Oxidative Stress, Antioxidant Capabilities, and Bioavailability: Ellagic Acid or Urolithins?. Antioxidants.

[61] Arrak J.K. (2010). Effect of Ellagic Acid Extracted from Pomegranate (*Punica granatum* L.) on Thyroid and Parathyroid Gland of Adult Rats Exposed to Lead Acetate. Kufa J. Vet. Med. Sci..

[62] Abd El-Emam M.M., Mostafa M., Farag A.A., Youssef H.S., El-Demerdash A.S., Bayoumi H., Gebba M.A., El-Halawani S.M., Saleh A.M., Badr A.M. (2023). The Potential Effects of Quercetin-Loaded Nanoliposomes on Amoxicillin/Clavulanate-Induced Hepatic Damage: Targeting the SIRT1/Nrf2/NF-κB Signaling Pathway and Microbiota Modulation. Antioxidants.

[63] Altamimi J.Z., AlFaris N.A., Alshammari G.M., Alagal R.I., Aljabryn D.H., Aldera H., Alrfaei B.M., Alkhateeb M.A., Yahya M.A. (2021). Ellagic acid protects against diabetic nephropathy in rats by regulating the transcription and activity of Nrf2. J. Funct. Foods.

[64] Wang Q., Botchway B.O.A., Zhang Y., Liu X. (2022). Ellagic acid activates the Keap1-Nrf2-ARE signaling pathway in improving Parkinson’s disease: A review. Biomed. Pharmacother..

[65] Wei Y.-z., Zhu G.-F., Zheng C.-Q., Li J.-J., Sheng S., Li D.-D., Wang G.-Q., Zhang F. (2020). Ellagic acid protects dopamine neurons from rotenone-induced neurotoxicity via activation of Nrf2 signalling. J. Cell Mol. Med..

[66] Chelombitko M.A. (2018). Role of Reactive Oxygen Species in Inflammation: A Minireview. Mosc. Univ. Biol. Sci. Bull..

[67] Chen P., Chen F., Zhou B. (2018). Antioxidative, anti-inflammatory and anti-apoptotic effects of ellagic acid in liver and brain of rats treated by D-galactose. Sci. Rep..

[68] Abd-Elhakim Y.M., Mohammed H.H., Mohamed W.A.M. (2018). Imidacloprid Impacts on Neurobehavioral Performance, Oxidative Stress, and Apoptotic Events in the Brain of Adolescent and Adult Rats. J. Agric. Food Chem..

[69] Harakeh S., Almuhayawi M., Jaouni S.A., Almasaudi S., Hassan S., Amri T.A., Azhar N., Abd-Allah E., Ali S., El-Shitany N. (2020). Antidiabetic effects of novel ellagic acid nanoformulation: Insulin-secreting and anti-apoptosis effects. Saudi J. Biol. Sci..

[70] Rostami A., Vakili S., Koohpeyma F., Jahromi B.N., Aghajari Z.A., Mahmoudikohani F., Saki F., Mahmoodi M., Jaberi K.R., Movahedpour A. (2022). Ellagic acid effects on testis, sex hormones, oxidative stress, and apoptosis in the relative sterility rat model following busulfan administration. BMC Complement. Med. Ther..

[71] Saeed Z.M., Khattab M.I., Khorshid N.E., Salem A.E. (2022). Ellagic acid and cilostazol ameliorate amikacin-induced nephrotoxicity in rats by downregulating oxidative stress, inflammation, and apoptosis. PLoS ONE.

[72] Xie W., Chen C., Li H., Tu Y., Zhong Y., Lin Z., Cai Z. (2024). Imidacloprid-induced lung injury in mice: Activation of the PI3K/AKT/NF-κB signaling pathway via TLR4 receptor engagement. Sci. Total Environ..

[73] Wu Z., Wang H., Fang S., Xu C. (2018). Roles of endoplasmic reticulum stress and autophagy on H_2_O_2_-induced oxidative stress injury in HepG2 cells. Mol. Med. Rep..

[74] Karim M.R., CFisher R., Kapphahn R.J., Polanco J.R., Ferrington D.A. (2020). Investigating AKT activation and autophagy in immunoproteasome-deficient retinal cells. PLoS ONE.

[75] Cheng Z. (2019). The FoxO-Autophagy Axis in Health and Disease. Trends Endocrinol. Metab..

[76] Deleyto-Seldas N., Efeyan A. (2021). The mTOR–Autophagy Axis and the Control of Metabolism. Front. Cell Dev. Biol..

[77] Wang Y., Ren F., Li B., Song Z., Chen P., Ouyang L. (2019). Ellagic acid exerts antitumor effects via the PI3K signaling pathway in endometrial cancer. J. Cancer.

[78] Yu J., Song S., Jiao J., Liu X., Zhu H., Xiu L., Sun D., Li Q., Yue X. (2020). ZiYinHuaTan Recipe Inhibits Cell Proliferation and Promotes Apoptosis in Gastric Cancer by Suppressing PI3K/AKT Pathway. BioMed Res. Int..

[79] Li X., Huang K., Liu X., Ruan H., Ma L., Liang J., Cui Y., Wang Y., Wu S., Li H. (2021). Ellagic Acid Attenuates BLM-Induced Pulmonary Fibrosis via Inhibiting Wnt Signaling Pathway. Front. Pharmacol..

[80] White E. (2016). Autophagy and p53. Cold Spring Harb. Perspect. Med..

[81] Park K.-J., Lee S.-H., Lee C.-H., Jang J.-Y., Chung J., Kwon M.-H., Kim Y.-S. (2009). Upregulation of Beclin-1 expression and phosphorylation of Bcl-2 and p53 are involved in the JNK-mediated autophagic cell death. Biochem. Biophys. Res. Commun..

[82] Maiuri M.C., Zalckvar E., Kimchi A., Kroemer G. (2007). Self-eating and self-killing: Crosstalk between autophagy and apoptosis. Nat. Rev. Mol. Cell Biol..

[83] Tran S., Fairlie W.D., Lee E.F. (2021). BECLIN1: Protein Structure, Function and Regulation. Cells.

[84] Baeeri M., Momtaz S., Navaei-Nigjeh M., Niaz K., Rahimifard M., Ghasemi-Niri S.F., Sanadgol N., Hodjat M., Sharifzadeh M., Abdollahi M. (2017). Molecular evidence on the protective effect of ellagic acid on phosalone-induced senescence in rat embryonic fibroblast cells. Food Chem. Toxicol..

[85] Cai C.-C., Ye L.-X., Zhu J.-H., Bai J.-J., Zeng S.-S., Chen S.-Q., Lin Z.-L. (2019). Ellagic acid attenuates hypoxic-ischemic brain injury by alleviating autophagy. Chin. J. Pathophysiol..

